# Should English Doctors Dispense?

**Published:** 1896-06-06

**Authors:** 


### The Hospital

AN INSTITUTIONAL JOURNAL OF

The Medical Sciences and Hospital Administration,

WITH

AN EXTRA NURSING SUPPLEMENT.

VOL. XX.—No. 506.] [JUNE 6, 1896.

### SHOULD ENGLISH DOCTORS DISPENSE?

In France, it is stated, there are not more than three thousand of the whole fifty or sixty thousand medical practitioners who dispense their own medicines. Even those would not be permitted by law to do their own dispensing but for the fact that there are no registered chemists in their various localities. The French are a very logical people, and with their natural love of clear distinction between things that differ they have separated the medical from the pharmaceutical art with as much precision as the guillotine separates a head from a body.

It will be allowed by all persons who think a little that the technique of a man's daily business, the actual work to which he puts his hands as well as his head, has a very powerful and permanent effect upon his personality in the course of years. How is it one can almost always recognise a doctor, even though he be a perfect stranger, by his general appearance? He does not wear highly specialised clothing like the clergyman. On the contrary, so far as his dress is considered, there is usually little to distinguish him from the lawyer, the civil servant, or the ordinary gentleman of no particular occupation. And yet, in probably as many as seven cases out of ten in which a guess should be hazarded as to a stranger's occupation, the experienced man of the world would be able to diagnose a doctor with unfailing certainty.

The doctor who dispenses his own medicines is, we hold, markedly influenced by the fact, and not influenced for the better.

There can hardly be any doubt that if medical men never had dispensed their own medicines they would not now begin. The making up of physic bottles, and the folding of them in neat wrappers and sealing their corks with ornamental tips of sealing-wax, are not quite the kind of duties which require a long and severe curriculum of study, or which such a curriculum specially qualifies a man for. These things are essentially of the shop; and though we do not say that a man of the shop cannot also be a man of scientific methods and philosophic ways of looking at his work, we do say that the shop and its ways are not conducive to scientific methods and philosophic habits of mind. Now the modern doctor, if he is to practise physic as modern science, and, indeed, the modern public, understands it, must be a man of scientific methods and philosophic habits of mind. Especially must this be the case with the general practitioner, whether of consultant or family status. The problems he is almost daily called upon to solve in diagnosis, prognosis, and treatment are of such a kind that if he does not apply to them a trained intellect and the methods and substances which science has recently placed, and still is placing, in his hands, he is little better than a deceiver of the people.

It is believed that dispensing is profitable, and that it keeps patients in touch with the doctor's surgery, rather than with the chemist's shop. That may be so; and yet we feel that the modern doctor, educated as he is, and resolute as he ought to be to practise scientifically or not at all, is not in his place behind a dispensing counter. It may be a counsel of perfection to urge that dispensing should be given up by the men of the modern school; but, even if it be, is it not the truth that every part of our calling is now carried out, if carried out according to the highest known methods of science, in obedience to counsels of perfection? In truth, the modern doctor knows no approved rules of this art at all except what are counsels of perfection.

The question, in its essence, is really one of medical progress. In its aspirations—and may we not also say in its achievements?—medicine is almost the most progressive of all the scientific arts. But medicine is not really progressive for the body of the people if no more than a few of the leading men in any one country are striving to promote the highest possible degree of common health. If it be the fact that large numbers of practitioners have little or no enthusiasm for progress, or are too mentally indolent to care for any more science than is required for the earning of "bread and butter," is it not all the more incumbent upon the medical journalist to strive to waken intelligence, to kindle enthusiasm, and so to quicken and strengthen the pulsations of the great heart of the medical organism that the rich blood of the new science may reach and revivify every remotest part? In short, medicine is being called upon to accept a new *rôle*, the *rôle* of scientific leadership among the people. That part we cannot play if we do not read, and if we do not acquire and preserve the mental habits of the studious person. The "shop" is fatal to such habits. It ought to be done away with. A cultured thinker is what the world now expects the doctor to be, and a cultured thinker he must be if progression, and not retrogression, is to be the note of the future. As we said at the outset, we do not wish to make more of this comparatively small matter than there is in it. Only we are sure that every medical man in the kingdom will agree with this proposition, namely, that if dispensing could be given up without risk it would be well to give it up.

